# Impact of the G84E variant on *HOXB13* gene and protein expression in formalin-fixed, paraffin-embedded prostate tumours

**DOI:** 10.1038/s41598-017-18217-w

**Published:** 2017-12-19

**Authors:** Liesel M. FitzGerald, Kelsie Raspin, James R. Marthick, Matt A. Field, Roslyn C. Malley, Russell J. Thomson, Nicholas B. Blackburn, Annette Banks, Jac C. Charlesworth, Shaun Donovan, Joanne L. Dickinson

**Affiliations:** 1 Menzies Institute for Medical Research, University of Tasmania, Hobart, TAS 7000 Australia; 2 Australian Institute of Tropical Health and Medicine, James Cook University, Cairns, Qld 4878 Australia; 3 Genome Informatics, John Curtin School of Medical Research, Australian National University, Canberra, ACT 2601 Australia; 4 Royal Hobart Hospital, Hobart, TAS 7000 Australia; 5 School of Medicine, University of Tasmania, Hobart, TAS 7000 Australia; 6 Western Sydney University, Sydney, NSW 2150 Australia; 7 South Texas Diabetes and Obesity Institute, School of Medicine, University of Texas Rio Grande Valley, Brownsville, Texas 78520 USA; 8 Hobart Pathology, Hobart, TAS 7000 Australia

## Abstract

The *HOXB13* G84E variant is associated with risk of prostate cancer (PCa), however the role this variant plays in PCa development is unknown. This study examined 751 cases, 450 relatives and 355 controls to determine the contribution of this variant to PCa risk in Tasmania and investigated *HOXB13* gene and protein expression in tumours from nine G84E heterozygote variant and 13 wild-type carriers. Quantitative PCR and immunohistochemistry showed that *HOXB13* gene and protein expression did not differ between tumour samples from variant and wild-type carriers. Allele-specific transcription revealed that two of seven G84E carriers transcribed both the variant and wild-type allele, while five carriers transcribed the wild-type allele. Methylation of surrounding CpG sites was lower in the variant compared to the wild-type allele, however overall methylation across the region was very low. Notably, tumour characteristics were less aggressive in the two variant carriers that transcribed the variant allele compared to the five that did not. This study has shown that *HOXB13* expression does not differ between tumour tissue of G84E variant carriers and non-carriers. Intriguingly, the G84E variant allele was rarely transcribed in carriers, suggesting that *HOXB13* expression may be driven by the wild-type allele in the majority of carriers.

## Introduction

Family history is a widely recognised risk factor for prostate cancer (PCa) with an estimated heritability of 58%, the highest of any cancer^1,2^. While approximately 33% of familial risk is explained by common, low penetrance variants, a study by Mancuso and colleagues estimates that as much as 42% of ‘missing’ heritability is likely to be explained by rare variants^3,4^. Several recent studies have applied next-generation sequencing (NGS) technologies to familial PCa datasets with the aim of identifying rare disease-causing variants^5–7^. In particular, a targeted NGS study of 94 familial PCa probands, with evidence of linkage to chr17q21, identified a rare non-synonymous mutation in *HOXB13* (G84E; rs138213197)^5^. Ewing and colleagues observed that the case carrier frequency was higher in a familial cohort, 4.7% (OR 68.1, p = 0.001), compared to a population cohort, 1.4% (OR 20.1, p < 0.001)^5^. Several studies have since replicated this finding in Caucasian familial and case-control populations and estimate the variant to be associated with a 4- to 8-fold increase in PCa risk, as well as with early-onset disease^8–14^.

In the normal prostate, the highly expressed HOXB13 transcription factor plays a key role in prostate development^15^. Notably, HOXB13 has been shown to interact with the androgen receptor (AR), a protein essential for prostate development and required for all stages of PCa growth^16^. Norris and colleagues (2009) demonstrated that *HOXB13* acts as both a repressor and coactivator of AR target genes; in target genes with an androgen-response element (ARE) the HOXB13:AR complex inhibits transcription, but in genes with a HOX element, the complex enhances transcription^16^. *HOXB13* has been reported to function as a growth promoter and growth suppressor in prostate cancer models, depending on factors such as tumour androgen sensitivity status and cellular localisation of the protein (reviewed in^17^). Therefore, the role of *HOXB13* in prostate tumour development remains unclear and the mechanism by which the *HOXB13* gene and, specifically, the G84E variant promotes prostate carcinogenesis, is largely unknown. Further analyses are required to determine whether the G84E variant causes a gain or loss of gene function, or increases PCa risk through other mechanisms.

The objective of this study was to investigate the functionality of the *HOXB13* G84E variant by examining transcription, translation, and possible epigenetic modification of the gene, in archival prostate tumour specimens obtained from several variant carrier and non-carrier cases identified in a Tasmanian familial PCa cohort.

## Results

### Identification of the HOXB13 G84E variant in a Tasmanian PCa cohort

**Figure 1:**
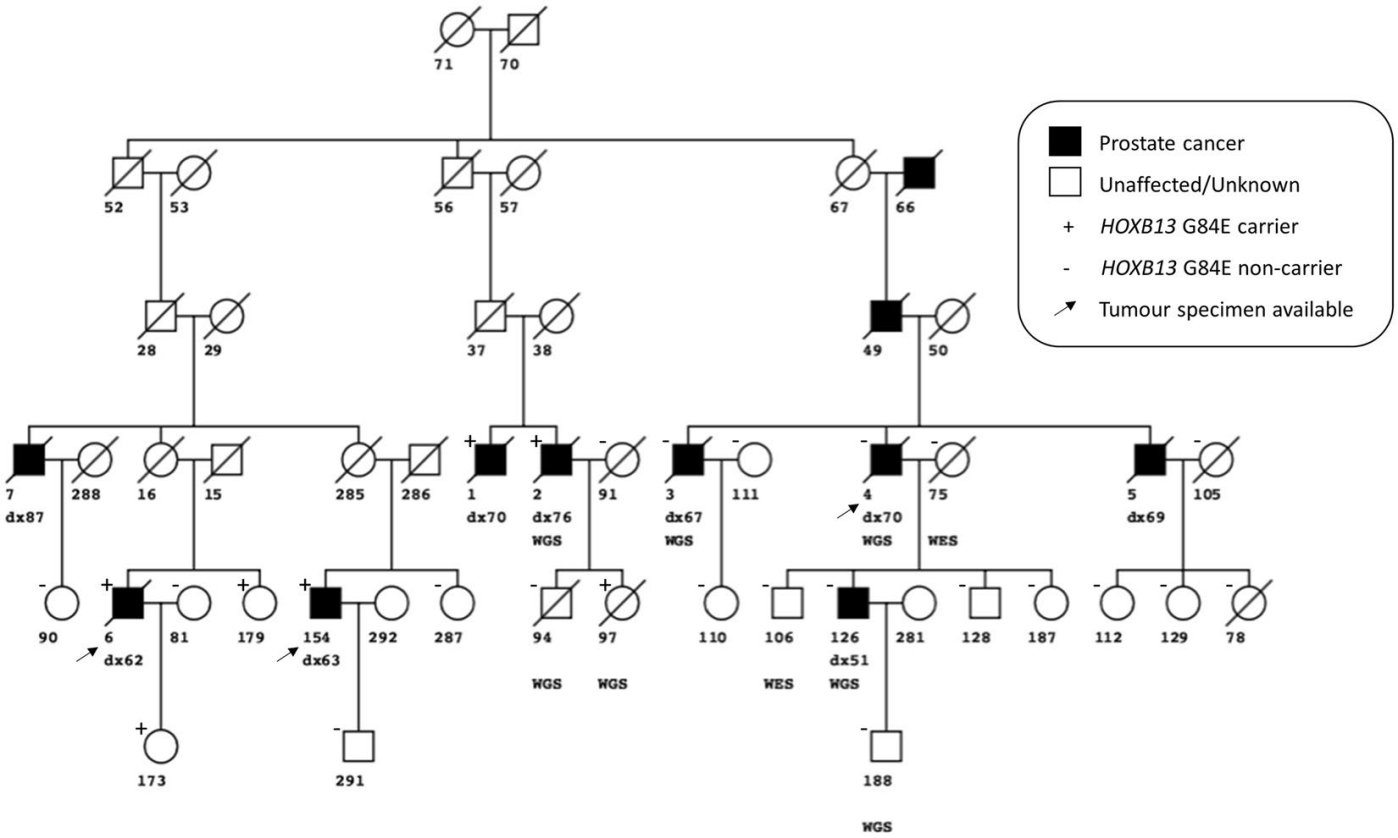
Segregation of the G84E variant in the familial prostate cancer pedigree, PcTas72. The G84E variant (+) was originally identified in individuals PcTas72–2 and PcTas72–97 using existing WGS data, with an additional five carriers identified in subsequent genotyping. Squares indicate males and circles females, with a slash indicating the subject is deceased. Individual identification numbers and age at diagnosis of cases are shown under each symbol. An arrow indicates a tumour specimen was obtained.

The G84E *HOXB13* variant was identified in existing whole-genome sequencing data obtained from a case and his daughter from a Tasmanian PCa family (Fig. 1; PcTas72–2, and PcTas72–97). Subsequent genotyping of all available PcTas72 family members (n = 61) confirmed the two carriers and identified a further five heterozygous carriers of the G84E variant, including three cases and two female relatives. Figure 1 shows segregation of the variant in cases from two branches of PcTas72 but not the third, underscoring the heterogeneity of this cancer, even within a single family.

### Association between PCa risk and the *HOXB13* G84E variant in the Tasmanian population

To assess the contribution of the G84E variant to PCa in the Tasmanian population, all individuals from the familial (n = 699) and case-control (n = 850) PCa cohorts were genotyped. A further 12 variant carriers were identified; six in five additional PcTas families (Supplementary Table 1), and four in the case-control cohort (three sporadic cases and one control). We also examined tumour tissue derived from pathology specimens for seven cases from the six variant carrier families, where germline DNA from blood or saliva was not available, and identified a further five carriers from two families (Supplementary Table 1). M_QLS_ analysis^18^ of the combined familial and case-control genotyping data demonstrated a significant association between PCa risk and the G84E variant in the Tasmanian population (OR = 6.59; p = 4.2 × 10^−5^) (Table 1). For the familial cases, the age at diagnosis and tumour grade of carriers in the six PcTas families were similar to non-carriers in these same families.

**Table 1:** M_QLS_ association analysis of G84E and prostate cancer risk in the combined familial and case-control Tasmanian datasets.

Familial Cohort - % G84E Carriers	Sporadic Cohort - % G84E Carriers	ExAC MAF (European/non-Finnish)	Odds Ratio
Cases – 3.23% Relatives – 1.34%	Cases – 0.62% Controls – 0.28%	0.28%	6.59 (p = 4.2 × 10^−5^)

### The effect of the G84E variant on *HOXB13* gene expression

**Figure 2:**
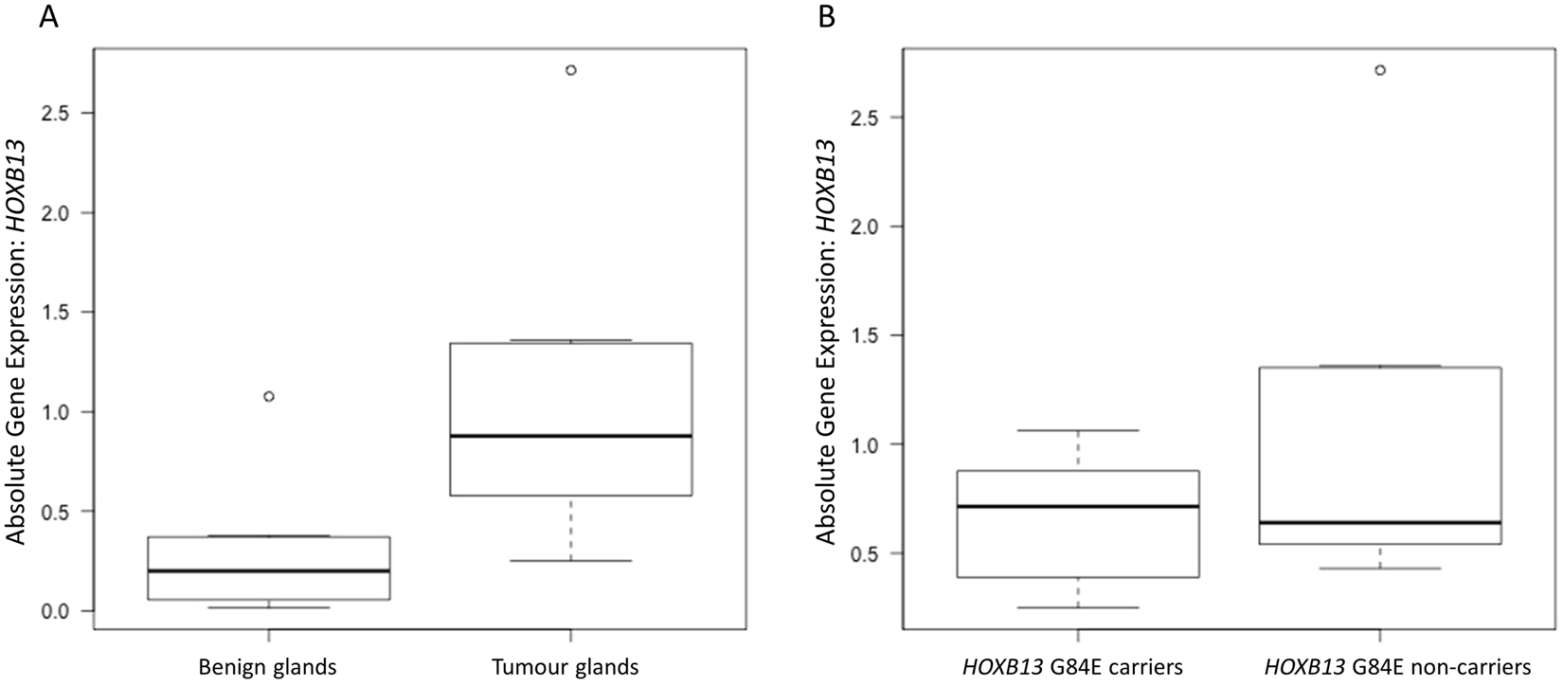
*HOXB13* gene expression analysis in FFPE prostate tumours. (**A**) A 1.5-fold increase in *HOXB13* expression was observed in malignant (n = 10) compared to benign cells (n = 10; p = 0.01). (**B**) No significant difference in *HOXB13* expression was observed between the tumour cells of variant carriers (n = 6) and non-carriers (n = 8; p = 0.21).

FFPE pathology blocks were obtained for 22 PcTas cases, 11 of which had G84E carrier status determined from a germline DNA sample (Table 2). Genotyping of prostate tissue DNA from the 22 blocks confirmed four and identified five additional heterozygous G84E carriers, including a case whose germline DNA was genotyped as wildtype (PcTas22–203; Table 2). Repeat genotyping of PcTas22–203 germline and re-extracted tumour DNA samples confirmed the discordant result. First-degree relatives of this individual were genotyped as G84E wildtype. No additional samples were available for this individual (deceased), therefore this anomaly could not be resolved to determine whether a pathology sample mix-up had occurred, mosaicism was present in the individual or the variant arose somatically. To investigate *HOXB13* gene expression, RNA was extracted from both adjacent benign and malignant cells for 10 cases. Due to the restricted availability of tissue, RNA was extracted from only benign cells for four cases, only malignant cells for four cases, and a mixed cell population for one case (Supplementary Table 2). *HOXB13* expression was initially investigated in the ten paired malignant-benign samples, where we observed significantly higher expression in malignant compared to benign cells (1.5-fold increase; p = 0.01; Fig. 2A). However, when *HOXB13* expression was compared between the malignant cells of G84E variant carriers (n = 6) and non-carriers (n = 8), there was no significant difference between the two (p = 0.21; Fig. 2B). There was also no detectable difference in *HOXB13* gene expression between the benign cells of variant carriers (n = 4) and non-carriers (n = 10; p = 0.29).

**Table 2:** Clinicopathological characteristics of FFPE prostate tissue samples obtained for *HOXB13* G84E carriers and non-carriers.

PcTas ID	Age at Diagnosis	Tissue Source	Germline Genotype	Tumour Genotype	Tumour Grade^1^	Contemporary Gleason Score^2^
PcTas4–3	80	TURP	CC	CC	M/PD	7 (4 + 3)
PcTas11–11	85	TURP	N/A	CC	—	7 (3 + 4)
PcTas11–12	58	TURP	N/A	CC	—	9 (4 + 5)
PcTas11–13	72	TURP	N/A	CC	—	No tumour
PcTas11–16	78	TURP	N/A	CC	—	5 (2 + 3)
PcTas12–1	63	RP	CC	CC	MD	6 (3 + 3)
PcTas12–6	80	TURP	N/A	CC	PD	7 (3 + 4)
PcTas12–9	68	TURP	N/A	CC	—	6 (3 + 3)
PcTas22–6	63	TURP	CC	CC	WD	No tumour
PcTas47–2	68	TURP	CC	CC	WD	No tumour
PcTas60–1	58	TURP	CC	CC	WD	6 (3 + 3)
PcTas63–24	67	TRUS	N/A	CC	MD	6 (3 + 3)
PcTas72–4	70	TURP	CC	CC	PD	9 (4 + 5)
PcTas12–3	62	TURP	N/A	CT	WD	4 (2 + 2)
PcTas12–7	59	TURP	N/A	CT	PD	9 (4 + 5)
PcTas12–8	73	TURP	N/A	CT	—	6 (3 + 3)
PcTas22–203	79	TRUS	CC	CT	PD	8 (4 + 4)
PcTas22–576	69	RP	N/A	CT	M/PD	7 (3 + 4)
PcTas22–637	70	TRUS	CT	CT	PD	8 (4 + 4)
PcTas72–6	62	TURP	CT	CT	W/MD	5 (3 + 2)
PcTas72–154	63	TRUS	CT	CT	WD	4 (2 + 2)
PcTas3250–1	51	RP	CT	CT	PD	9 (4 + 5)

^1^Tumour grade obtained from pathology report.

^2^Contemporary Gleason Score from FFPE tissue block chosen for macrodissection of nucleic acids and IHC. N/A: germline sample not available. WD: well differentiated. MD: moderately differentiated; PD. poorly differentiated. -: Information not present in original pathology report.

We next examined whether the variant transcript was detectable in the tumour tissue of seven G84E carriers. Next-generation sequencing applied to cDNA from freshly cut FFPE sections indicated that only two of seven variant carriers (28.6%) showed evidence of variant allele transcription (Supplementary Table 2). The variant transcript was detectable in both malignant and benign cells in one individual and in benign cells only in the second individual.

To determine whether imbalanced allele transcription was related to *HOXB13* G84E carrier status, allele-specific transcription was determined for another variant in relatively close proximity to the G84E variant. The *HOXB13* variant, rs9900627 (MAF 11.2%), is 262 bp centromeric to G84E in exon 1, and one heterozygous carrier of this variant was identified in our tissue cohort (PcTas11–11; G84E negative). Unlike carriers of G84E, the variant and wild-type alleles of rs9900627 were detectable in equal proportions in PcTas11–11.

### The effect of the G84E variant on HOXB13 protein expression

**Figure 3:**
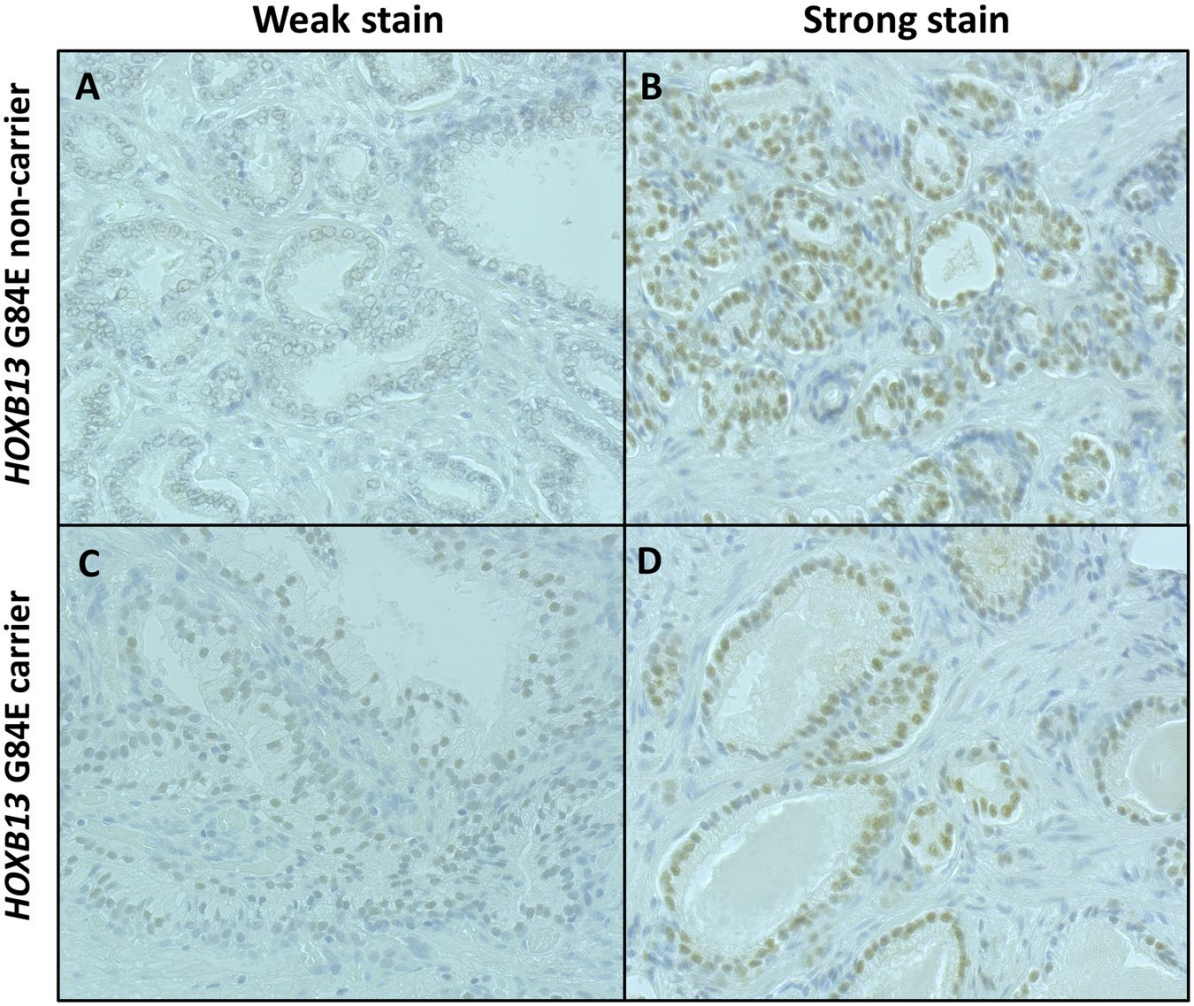
HOXB13 protein expression in G84E non-carriers (**A** and **B**) and carriers (**C** and **D**). Immunohistochemistry staining intensity of HOXB13 was not consistent with G84E carrier status (**A** and **C)** weak staining; (**B** and **D)** strong staining). HOXB13 was predominantly expressed in the nuclei of prostate gland cells, while cytoplasmic cells were often negative or only weakly positive for the protein. Images were taken on a Leica 2500 microscope (x200) using the Lieca Application Suite V3.

Immunohistochemistry was undertaken on all 22 FFPE pathology samples to determine whether protein expression differed between malignant and benign prostate tissue, and between *HOXB13* variant and non-variant carriers. HOXB13 staining ranged from weak to strong across the dataset (Fig. 3; Supplementary Table 2). Analyses of the quasi-continuous nuclear scores from 16 samples with paired malignant and benign cells did not reveal any significant difference in HOXB13 protein expression between malignant and benign cells (p = 0.45). Analysis of malignant cells from G84E variant carriers (n = 9) *versus* non-carriers (n = 9) also indicated no significant difference between the two groups (p = 0.68). A similar result was observed for benign cells (p = 0.84).

### Methylation of HOXB13 CpG islands

**Figure 4:**
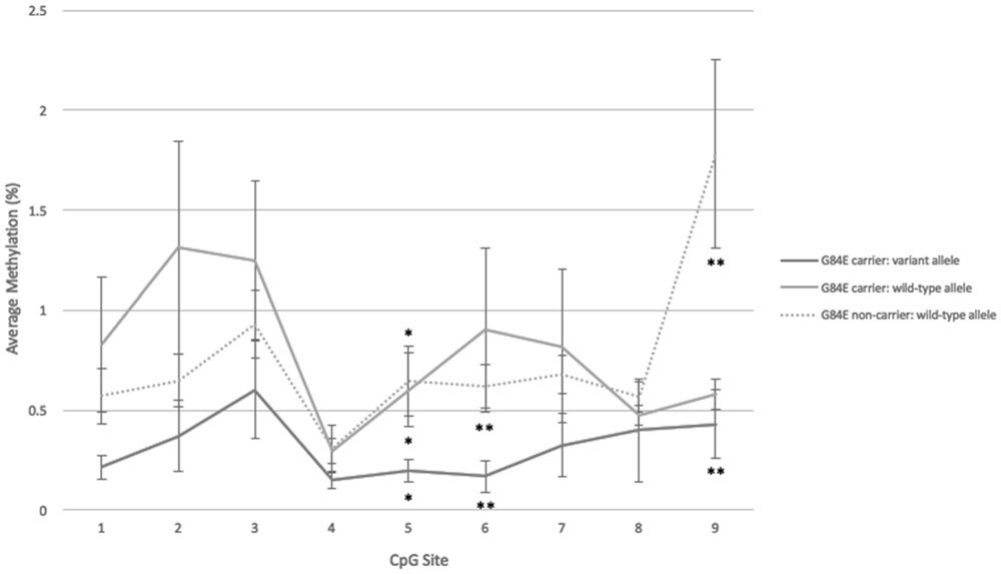
Average methylation (%) across nine CpG sites located within a CpG island surrounding the G84E variant. Average methylation for both carriers and non-carriers was low across all CpG sites. The variant allele was less methylated at all sites (solid black line) when compared to the wild-type allele of both variant carriers (grey solid line) and non-carriers (dotted line). The difference in methylation was statistically significant between all alleles at CpG site 5, and between the variant allele and the non-carrier wildtype allele at CpG sites 6 and 9 (*p < 0.05, **p < 0.001).

We next examined DNA methylation in tumour samples at two *HOXB13* CpG islands, one spanning the promoter region and exon 1 of the gene, and the second located ~4.5 kb upstream of the *HOXB13* transcription start site (Supplementary Figure 1). Allele-specific methylation was also examined at nine CpG sites within the promoter/exon 1 CpG island and surrounding the G4E variant, to determine if differential methylation explained the observed unbalanced allele transcription. Very low levels of DNA methylation were observed across both CpG islands in both variant (n = 3) and non-variant carriers (n = 3; Supplementary Figure 2). Allele-specific methylation was consistently low across all nine CpG sites within the promoter/exon 1 CpG island in both variant carriers (n = 7) and non-carriers (n = 7); however, methylation of the variant allele was lower than that of the wild-type allele in all instances (Fig. 4). Significant differences in CpG site-specific methylation between the variant allele and wild-type alleles of carriers and/or non-carriers was observed at three CpG sites (p < 0.05; Fig. 4), while no difference was observed between the wild-type alleles of carriers and non-carriers. No correlation between methylation and transcription of the variant allele, or absolute gene expression of *HOXB13* was observed (data not shown).

## Discussion

The *HOXB13* G84E variant is a PCa risk variant reported to be of particular relevance to early onset familial disease^5,19,20^. Here, a Tasmanian PCa family was shown to be segregating the *HOXB13* variant, which was subsequently demonstrated to be significantly associated with PCa risk in the Tasmanian population (OR = 6.59). While the *HOXB13* gene is known to play a key role in prostate development^15^ and to interact with the AR to influence transciption^16^, little is known about how the *HOXB13* G84E variant causes increased PCa risk.

A computational modeling study by Chandrasekaran *et al*. (2017) has suggested that the G84E variant increases HOXB13 protein stability^21^, which may in turn cause increased transcription of downstream target genes promoting cell proliferation and invasion^22^. However, in an *in vitro* cell model study using site directed mutatgenesis, Cardoso and colleagues (2016) found that the G84E variant had no phenotypic impact, i.e. there was no change in proliferation or apoptosis compared to the wild-type cell model^23^. In our study of FFPE prostate tumour tissue, we found no difference in HOXB13 protein expression between G84E carriers and non-carriers; a finding supported by a larger IHC study of radical prostatectomy samples from 101 G84E carriers and 99 non-carriers^24^. Furthermore, we demonstated that gene expression was comparable between carriers and non-carriers. Although tumour tissue samples from carriers were demonstrated to be heterozygous for the G84E variant, the variant allele transcript was rarely detectable in G84E carrier prostate tissue (benign or malignant). In fact, the variant allele was only detectable in two of seven carriers and at lower levels than the wild-type allele. To further examine *HOXB13* allelic expression, transcription of another *HOXB13* variant (rs9900627) in close proximity to G84E was examined. Comparable transcription of both the wild-type and variant rs9900627 alleles was evident in the tumour tissue of a non-G84E carrier. We therefore hypothesize that the unbalanced allele transcription may be related to the presence of the G84E variant. Unbalanced allele transcription has been reported previously in a study of breast cancer patients^25^. Benz *et al*. (2006) investigated the common *ERBB2* variant, G1170C, in *ERBB2*-positive and *ERBB2*-negative breast cancer patients and found that *ERBB2*-positive germline heterozygotes had markedly skewed tumour genotype frequencies to homozygosity of either allele and monoallelic transcript amplification^25^. While this was not unexpected given previous research^26,27^, when the tumours of *ERBB2*-negative germline heterozygotes were examined, Benz *et al*. found that although tumour genotyping supported the heterozygous state, similar to our study, 70% of tumours showed preferential transcription of one allele, or unbalanced allele transcription. The authors suggested the unbalanced allele transcription in *ERBB2*-negative tumours may be due to epigenetic mechanisms, whereby methylation silences a particular allele.

Two CpG islands are located within or near the *HOXB13* gene: the first spans the promoter and exon 1 region of the gene and the second is ~4.5 kb upstream of the *HOXB13* transcription start site^28^. In a study of colorectal cancer, Ghoshal and colleagues (2010) found very little methylation in the promoter/exon 1 CpG island in both tumour and normal cell lines, whilst the upstream CpG island was significantly more methylated in tumour compared to normal cell lines^28^. Furthermore, they found that hypermethylation of the upstream CpG island partially supressed *HOXB13* expression and speculate that this region may function as an enhancer. In our study, we observed very low levels of DNA methylation at both CpG islands in all tumour samples tested (G84E carriers and non-carriers). When we looked further at allele-specific methylation of nine CpG sites surrounding the G84E variant in exon 1, although methylation was lower at three CpG sites on the variant allele, the overall level of methylation across this region was again so low that the biological impact this would have on gene transcription is questionable. It therefore seems unlikely that methylation differences explain the unbalanced allele transcription we observe in the G84E carriers. Alternatively, copy number variation at the *HOXB13* site or rapid targeted degradation of the variant mRNA transcript may underpin the observed allelic imbalance and warrants further investigation.

Interestingly, while the numbers were too small for formal analyses, examination of clinical characteristics revealed that *HOXB13* G84E variant carriers that transcribed the variant allele (PcTas12–3 and PcTas72–6) had lower tumour grades and Gleason scores (GS) than variant carriers that did not transcribe the variant allele. The tumours from both PcTas12–3 and PcTas72–6 were well- to moderately-differentiated with GS < 6, whereas where there was no evidence for variant allele transcription, the tumours were predominantly poorly differentiated with GS ≥ 7, with the exception of one sample (PcTas12–8; GS6). Due to insufficient tumour material, allele-specific transcription was not able to be determined for two variant carriers, PcTas22–637 and PcTas72–154, who had a poorly differentiated and well differentiated tumour grade respectively. Several previous studies have investigated possible associations between G84E and clinicopathological factors, and the majority have found no association between carrier status and GS^5,11,29,30^. However, two studies have presented contrary results. A Danish study of 995 patients (25 carriers) found G84E carrier status was significantly associated with GS ≥ 7 versus GS < 7^31^, whilst another study of 1,457 patients (18 carriers) observed that the G84E variant was more strongly associated with GS ≥ 7(4 + 3) disease but this was not significantly different to the association with GS ≤ 7(3 + 4) disease^13^. While it has to again be noted that our observations are based on limited numbers, these conflicting results may be due to the underlying variability in variant allele transcription that we have observed and this should be explored in a larger *HOXB13* G84E tumour dataset.

This study has provided important insights into the effect the *HOXB13* variant has on gene transcription in prostate tumour tissue but there are some limitations in the interpretation of data. Due to the rarity of the variant and the limited availability of informative tumour tissue specimens, the number of samples available for G84E variant carriers was restricted. The quality of DNA and RNA extracted from FFPE tissues is also fairly poor, therefore it is important that our findings are validated in larger FFPE cohorts or, if available, fresh frozen samples. In our IHC experiment, the antibody used was not specific to the variant form of the HOXB13 protein and it would be valuable to verify our gene expression results with a variant-specific protein antibody.

In summary, this study has demonstrated that *HOXB13* gene and protein expression do not differ in the tumours of G84E carriers and non-carriers. We have also demonstrated that in our tumour population, only a minority of variant carriers transcribe the G84E variant allele but the mechanism behind this unbalanced allele transcription is still unknown. Furthermore, we observed that clinicopathological features tended to be less aggressive in the tumours of carriers that did transcribe the variant allele. It is important that these findings are validated in a larger tissue cohort of *HOXB13* G84E carriers.

## Methods

### Study Resource

#### Tasmanian Prostate Cancer Familial Cohort

This cohort is comprised of a rare collection of 52 PCa families from the founder population of Tasmania. The number of affected men in these families range from five to over 140, and include up to five affected brothers and multiple father/son and uncle/nephew pairs. DNA samples from blood and saliva have been collected for 249 affected men and 450 male and female relatives. Archived prostate tissue pathology blocks from *HOXB13* G84E variant carriers and a random selection of non-carriers were targeted for collection from pathology labs. Sectioned FFPE pathology blocks were histologically reviewed by RM and SD to provide a contemporary grading of the tumours and to mark regions of malignant and benign cells.

#### Tasmanian Case-Control Cohort

The Tasmanian *Case-Control Prostate Cancer Study* is a population-based cohort, which includes blood or saliva samples from 495 cases and 355 controls. Cases were identified from the Tasmanian Cancer Registry (TCR) and considered eligible for this study if they were diagnosed under the age of 75 between the years 1996 and 2005. Controls were selected at random from the Tasmanian electoral roll and frequency matched by five year age groups to the cases. Controls are periodically checked against the TCR for PCa diagnosis.

Ethics approval for both cohort studies was obtained from the Human Research Ethics Committee Tasmania, Australia (H9999 and H007740) and written informed consent was obtained from all participating individuals. For deceased familial cases, a waiver of consent was obtained to collect prostate tissue specimens.

### Nucleic Acid Extractions

DNA was extracted from blood using the Nucleon BACC3 Kit (GE Healthcare) and from saliva using the Oragene DNA Kit (DNA Genotek) according to the manufacturers’ directions. FFPE tissue blocks were sectioned to 8 μm, dewaxed and rehydrated using a standard xylene-ethanol deparaffinisation protocol. Tumour and benign glands were marked on haematoxylin and eosin stained tissue sections by a pathologist (SD, RM). Marked tumour and benign regions were macro-dissected separately for both DNA and RNA. DNA was extracted using the QIAamp DNA FFPE Tissue Kit (QIAGEN) according to the manufacturer’s instructions and eluted in 50 μL of ATE Buffer. DNA was quantified using the Nanodrop® ND-1000 UV-vis spectrophotometer (Nanodrop® Technologies). RNA was extracted using the RecoverAll Total Nucleic Acid Isolation Kit (ThermoFisher Scientific) according to the manufacturer’s instructions and eluted in 30  μL of dH_2_O. RNA quality and quantity was assessed using the 2100 Bioanalzyer (Agilent). The SuperScript^TM^ VILO^TM^ cDNA Synthesis Kit (Invitrogen) was used for cDNA conversion, as per the manufacturer’s instructions.

### Next-Generation Sequencing Data

Existing whole-genome and whole-exome sequencing data was previously generated for several PcTas72 individuals (Fig. 1) at the Illumina Genome Network, USA, on the HiSeq 2500 s or the Kinghorn Centre for Clinical Genomics, Australia, on the Illumina HiSeq X^TM^ Ten platform using the TruSeq Nano library preparation. Data had been analysed using the Variant Analysis of Sequenced Pedigrees (VASP) analytical pipeline, developed specifically to detect disease causing variants in sequenced pedigrees^32,33^. The presence of the *HOXB13* G84E variant was examined in the annotated variant list generated using the Ensembl Variant Effect Predictor and overlapped with Ensembl canonical transcripts.

### Sanger Sequencing

Sanger sequencing was performed to validate the WGS results in the original PcTas72 carriers, as well as determine the *HOXB13* variant carrier status of deceased affected men who only had a tumour specimen available for genotyping. *HOXB13* primers were designed using Primer3Plus^34,35^ (Supplementary Table 3) and PCR conditions are available on request.

### Genotyping

A TaqMan SNP genotyping assay (Applied Biosystems) was used to genotype the *HOXB13* G84E variant in the familial and case-control cohorts on the LightCycler® 480 system (Roche). Heterozygous individuals were confirmed by Sanger sequencing, as described above.

### Real-Time qPCR Analysis

SYBR green real-time quantitative PCR (qPCR) assays were performed to determine gene expression of *HOXB13* and two housekeeping genes, *GAPDH* and *β-Actin*. Amplification was performed on 50ng FFPE cDNA using published *HOXB13*, *GAPDH* and *β-Actin* primers (Integrated DNA Technologies; Supplementary Table 3)^36,37^. Standard curves were generated for *HOXB13*, *GAPDH*, and *β-Actin* to determine PCR efficiency and normalise absolute *HOXB13* gene expression.

### MiSeq cDNA Sequencing

Transcription of the wild-type and mutant alleles of *HOXB13* were determined using the MiSeq platform (Illumina). A 157 bp region of *HOXB13*, covering the G84E mutation, was FFPE cDNA amplified and barcoded with unique forward and reverse tags (Supplementary Table 3). Barcoded DNA fragments were pooled, purified and libraries were sequenced on the Illumina MiSeq platform using the MiSeq® V2 300 Cycle Reagent Kit (Illumina).

FASTQ files were aligned to the hg19 reference genome using Galaxy version 16.04 (usegalaxy.org)^38–40^. The raw sequence data (FASTQ files) were converted to Sanger and Illumina 1.8+ format using the FASTQ Groomer tool, followed by realignment using BWA-MEM. The allele frequency at the variant position was visualised using IGV 2.3.68^41^. FastQC of BAM files was used to assess the quality of the raw sequence data.

### Methylation Analysis

FFPE DNA (~200 ng) was bisulphite converted using the EZ DNA Methylation-Gold^TM^ Kit (Zymo Research Corp). Two primer sets were designed to amplify fragments covering the HOXB13 promoter/exon 1 CpG island and a CpG island ~4.5 kb upstream of the transcription start site (Supplementary Figure 1; Supplementary Table 3). Fragments were purified using the QIAquick Gel Extraction Kit (Qiagen), as per the manufacturer’s instructions, and cloned in to the p-GEM®-T Easy Vector Kit (Promega Corporation), using a 3:1 ratio of insert to vector. Top10 competent cells (Invitrogen) were transformed with 2 µL of ligations. Ten white clones per sample were selected for amplification and DNA extraction, using the QIAprep Spin Miniprep Kit (Qiagen), as per the manufacturer’s instructions. Clone inserts were sequenced using the reverse *Sp6* primer. Each clone CpG site was scored as either 1 (methylated) or 0 (non-methylated), and bubble maps were generated using the CpG Bubble Chart Generator, Version 20061209 Alpha, created by Mark A Miranda.

Allele-specific methylation involved the PCR amplification of a 175 bp region of *HOXB13*, including the G84E mutation and nine surrounding CpG sites, using bisulphite-converted FFPE DNA, as described above (Supplementary Figure 1; Supplementary Table 3). Products were barcoded with unique forward and reverse tags and sequenced on the Illumina MiSeq platform, as described above. FASTQ files were quality score checked and separated into reads containing the G84E variant allele or the wild-type allele. A beta value (β), the ratio of methylated versus non-methylated reads, was determined for all of the nine CpG sites.

### Immunohistochemistry

Tissue sections (3.5 μm) were pre-treated with Target Retrieval Solution (Dako), followed by inactivation of endogenous peroxidases using 3% hydrogen peroxidase (Sigma-Aldrich). Non-specific staining was blocked using Protein Block (Dako). Sections were incubated with primary HOXB13 antibody (sc-28333; Santa Cruz Biotechnology; working dilution 1:50) in a humidified chamber for one hour, followed by 30 min incubation with an anti-mouse HRP-Labelled Polymer (Dako). *HOXB13* protein staining was visualised with 3–3′ diaminobenzidine (DAB) for 10 min, and the sections were counterstained using Mayer’s haematoxylin. Normal prostate glands (Abcam), ascertained as *HOXB13* wildtype by Sanger sequencing, were used as positive controls for the immunohistochemical reactions. Negative controls included primary antibody only, secondary antibody only, and a mouse IgG_1_ isotype control (Dako).

The immuno-stained sections were scored by a pathology registrar (RM) blinded to the *HOXB13* carrier status. Nuclear staining was scored as none/weak, moderate or strong, depending on the most common staining intensity in the entire tissue section. Immunostaining was assessed using a quasi-continuous nuclear score, created by multiplying each intensity level (1 for no/weak stain, 2 for moderate stain, and 3 for intense stain) by the corresponding percentage of positive cells. As benign prostate tissue was also present in some sections, immunostaining was assessed for both tumour and benign cells separately.

### Statistical Analyses

#### Association analyses

*HOXB13* genotype data was analysed using M_QLS_^18^, an association analysis that maximises power by performing tests of association in the combined familial and case-control datasets, while taking into account relatedness of individuals. M_QLS_ uses variance components to examine the significance of association for related individuals, and when the disease status is known for first-degree relatives of cases, M_QLS_ obtains more power by giving increased weighting to those individuals with closely related disease-carrying relatives.

#### Gene expression

The paired Student’s t-test was used to compare absolute *HOXB13* gene copy numbers between tumour and adjacent benign cells. The unpaired Student’s t-test was used to compare absolute *HOXB13* gene copy numbers in the tumour cells of G84E variant carriers *versus* non-carriers, and in the benign cells of G84E variant carriers *versus* non-carriers. P values < 0.05 were considered to be significant. Fold changes were also presented in box plot format using R version 3.4.0.

#### Protein expression

The paired Student’s t-test was used to compare HOXB13 protein expression between tumour and adjacent benign cells. Unpaired Student’s t-tests were used to compare HOXB13 protein expression in the tumour cells of G84E variant carriers *versus* non-carriers, and in the benign cells of G84E carriers versus non-carriers. P values < 0.05 were considered to be significant.

#### Methylation

Unpaired Student’s t-tests were used to compare methylation (β value) in reads containing the G84E variant allele versus reads with the wild-type. P values < 0.05 were considered to be significant.

### Data Availability

The data generated during this study are either available in the Supplementary Materials section or from the corresponding author on reasonable request.

## Electronic supplementary material

Supplementary Material
