# Supplementary material for: Impact of the G84E variant on HOXB13 gene and protein expression in formalin-fixed, paraffin-embedded prostate tumours
Source: Sci Rep. 2017 Dec 19;7:17778. doi: 10.1038/s41598-017-18217-w (PMC5736598; doi:10.1038/s41598-017-18217-w)
Supplement: Supplementary file 1 — Supplementary Material [file 41598_2017_18217_MOESM1_ESM.doc]

**Impact of the G84E variant on *HOXB13* gene and protein expression in formalin-fixed, paraffin-embedded prostate tumours**

Liesel M FitzGerald^1,†^, Kelsie Raspin^1,†^, James R Marthick^1^, Matt A Field^2,3^, Roslyn C Malley^4,5^, Russell J Thomson^6^, Nicholas B Blackburn^7^, Annette Banks^1^, Jac C Charlesworth^1^, Shaun Donovan^8^, and Joanne L Dickinson^1,*^

^1^ Menzies Institute for Medical Research, University of Tasmania, Hobart, TAS, 7000, Australia

^2^ Australian Institute of Tropical Health and Medicine, James Cook University, Cairns, Qld, 4878, Australia

^3^ Genome Informatics, John Curtin School of Medical Research, Australian National University, Canberra, ACT, 2601, Australia

^4^ Royal Hobart Hospital, Hobart, TAS, 7000, Australia

^5^ School of Medicine, University of Tasmania, Hobart, TAS, 7000, Australia

^6^ Western Sydney University, Sydney, NSW, 2150, Australia

^7^ South Texas Diabetes and Obesity Institute, School of Medicine, University of Texas Rio Grande Valley, Brownsville, Texas, 78520, USA

^8^ Hobart Pathology, Hobart, TAS, 7000, Australia

**
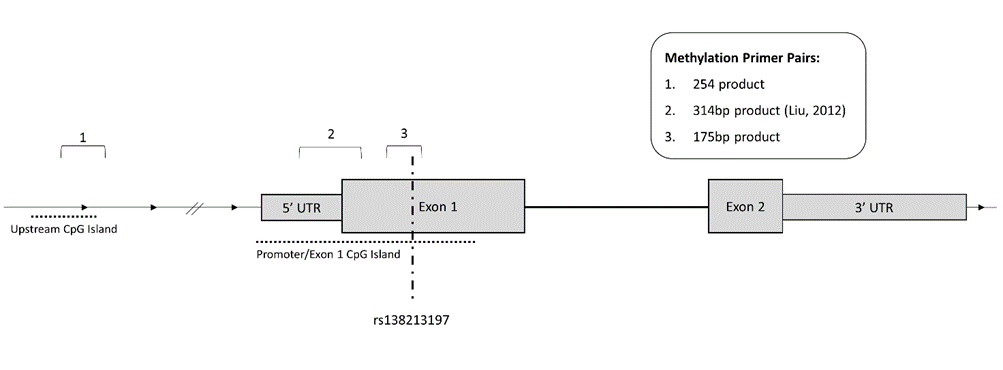
**

**Supplementary Figure 1**. Schematic of the *HOXB13* gene, indicating specific primer pairs used to analyse DNA methylation at two CpG islands; one spanning the promoter and exon 1 region and the other located ~4.5kb upstream of the *HOXB13* transcription start site. The location of the *HOXB13* G84E variant is marked with a dashed line. Primer sequences are shown in Supplementary Table 3. Please note, this diagram is not to scale.

**
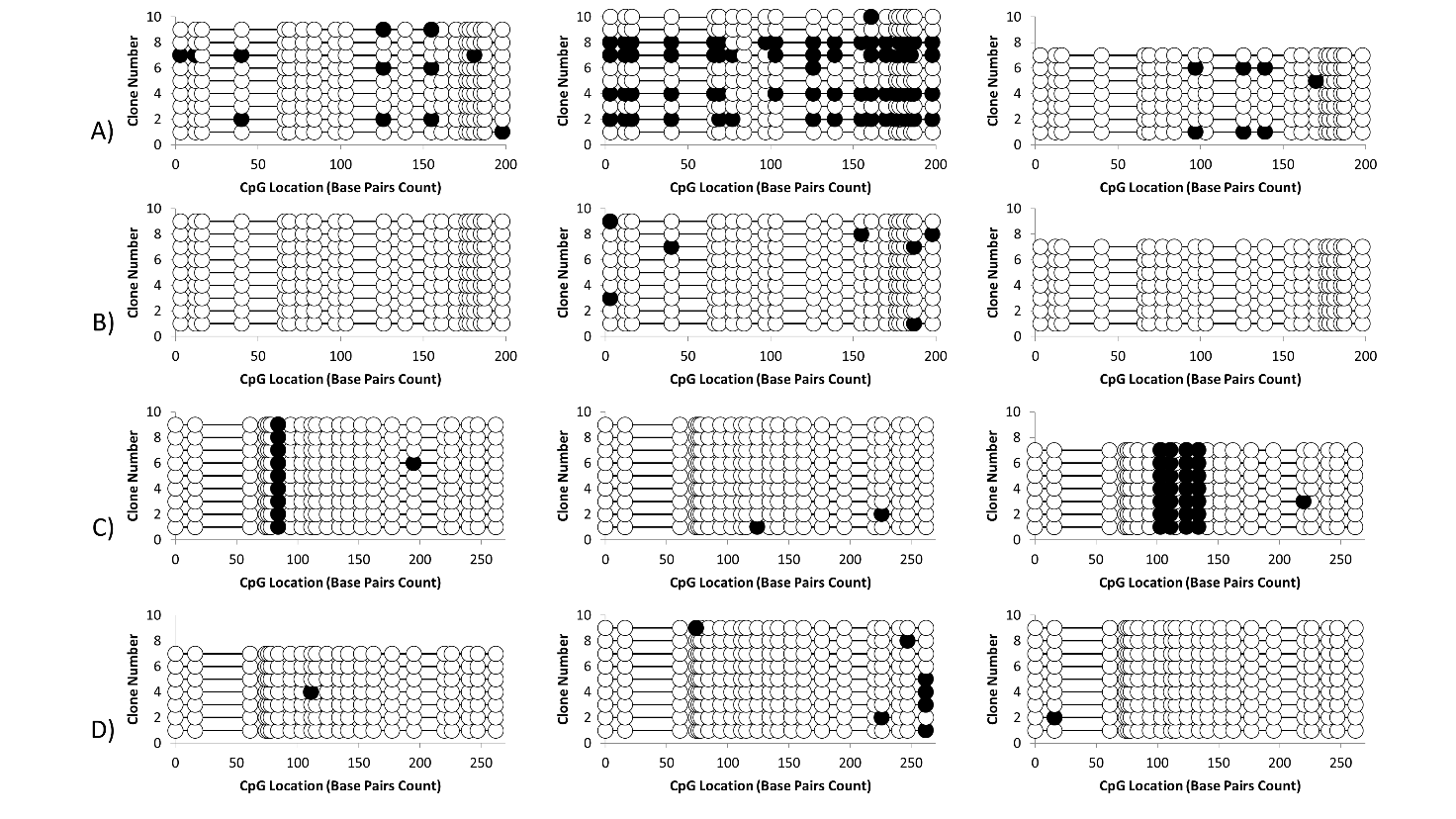
**

**Supplementary Figure 2.** Bubble maps showing methylation patterns across the two *HOXB13* CpG islands of interest in G84E non-carriers (A and C) and carriers (B and D). Open circles indicate non-methylated CpG sites while coloured circles indicate methylated sites. Very low levels of FFPE DNA methylation were observed in the CpG island upstream of the *HOXB13* transcription start site in both G84E non-carriers (A; n=3) and carriers (B; n=3). This pattern was similar in the promoter/exon 1 CpG island for non-carriers (C; n=3) and carriers (D; n=3). Bubble maps were produced using CpG Bubble Chart Generator, Version 20061209 Alpha.

**Supplementary Table 1: Summary of *HOXB13*** G84E variant carriers in PcTas families

|  | **Available Samples** | | | ***HOXB13* G84E Carriers (Case/Relative)** | | | |
| --- | --- | --- | --- | --- | --- | --- | --- |
| **PcTas Family** | **Germline** | **Tumour** | **Total** | **WES** | **Germline** | **Tumour** | **Total** |
| PcTas72 | 61 | 3 | 61 | 1/1 | 4/3 | 2 | 4/3 |
| PcTas12 | 50 | 6 | 54 |  | 0/2 | 3 | 3/2 |
| PcTas22 | 97 | 4 | 99 |  | 1/0 | 3^1^ | 3/0 |
| PcTas63 | 38 | 1 | 39 |  | 0/1 | 0 | 0/1 |
| PcTas213 | 23 | 0 | 23 |  | 1/0 | 0 | 1/0 |
| PcTas3250 | 1 | 1 | 1 |  | 1/0 | 1 | 1/0 |
| ^1^Includes PcTas22-203 | | | | | | | |

**Supplementary Table 2: Raw data from gen**e and protein expression analysis of FFPE prostate tissue specimens

| **PcTas ID** | **Tissue Cell Type** | **Gene Expression** | **G84E Variant Allele Transcription^1^** | **IHC Score** | **Protein Expression Final Score** | **Methylation Assays^2^** |
| --- | --- | --- | --- | --- | --- | --- |
| PcTas4-03 | Malignant | 1.36 |  | 2 (69%) | 1.38 | 1, 2, 3 |
|  | Benign | 0.02 |  | 3 (83%) | 2.49 |  |
| PcTas11-11 | Malignant | 0.59 |  | 3 (94%) | 2.82 | 1, 2, 3 |
|  | Benign | 0.17 |  | 2 (88%) | 1.76 |  |
| PcTas11-12 | Malignant | 0.50 |  | 3 (98%) | 2.94 |  |
|  | Benign |  |  | 2 (59%) | 1.18 |  |
| PcTas11-13 | Malignant |  |  |  |  |  |
|  | Benign | 0.44 |  | 3 (89%) | 2.67 | 3 |
| PcTas11-16 | Malignant |  |  |  |  |  |
|  | Benign | 0.23 |  | 3 (79%) | 2.37 | 2, 3 |
| PcTas12-01 | Malignant | 2.72 |  | 1 (77%) | 0.77 |  |
|  | Benign | 0.38 |  | 1 (34%) | 0.34 |  |
| PcTas12-06 | Malignant | 0.69 |  | 2 (81%) | 0.62 | 3 |
|  | Benign |  |  | 2 (72%) | 1.44 |  |
| PcTas12-09 | Malignant | 0.43 |  | 2 (92%) | 1.84 | 1, 3 |
|  | Benign | 0.37 |  | 3 (93%) | 2.79 |  |
| PcTas22-06 | Malignant |  |  |  |  |  |
|  | Benign | 0.42 |  | 3 (91%) | 2.73 |  |
| PcTas47-02 | Malignant |  |  |  |  |  |
|  | Benign | 0.30 |  | 1 (55%) | 0.55 |  |
| PcTas60-01 | Malignant | 0.58 |  | 2 (80%) | 1.6 |  |
|  | Benign | 0.02 |  | 2 (59%) | 1.18 |  |
| PcTas63-24 | Malignant |  |  | 2 (100%) | 2 |  |
|  | Benign |  |  |  |  |  |
| PcTas72-04 | Malignant | 1.34 |  | 2 (88%) | 1.76 | 3 |
|  | Benign | 0.19 |  | 1 (74%) | 0.74 |  |
| PcTas12-03 | Malignant | 0.25 | + | 3 (90%) | 2.7 | 1, 2, 3 |
|  | Benign | 0.05 | + | 3 (80%) | 2.4 | 3 |
| PcTas12-07 | Malignant | 0.40 | - | 2 (91%) | 1.82 | 1, 2, 3 |
|  | Benign |  |  |  |  |  |
| PcTas12-08 | Malignant | 0.38 | - | 1 (69%) | 0.69 |  |
|  | Benign |  |  | 2 (77%) | 1.54 | 3 |
| PcTas22-203 | Malignant |  | -^3^ | 2 (69%) | 1.38 | 1, 2, 3^3^ |
|  | Benign |  |  | 2 (65%) | 1.3 |  |
| PcTas22-576 | Malignant | 0.97 | - | 3 (93%) | 2.79 | 3 |
|  | Benign | 0.21 | - | 1 (72%) | 0.72 | 3 |
| PcTas22-637 | Malignant |  |  | 3 (80%) | 2.4 |  |
|  | Benign |  |  | 3 (78%) | 2.34 |  |
| PcTas72-06 | Malignant | 0.78 | - | 3 (91%) | 2.73 | 3 |
|  | Benign | 0.37 | + | 3 (81%) | 2.43 | 3 |
| PcTas72-154 | Malignant |  |  | 2 (81%) | 1.62 |  |
|  | Benign |  |  | 3 (80%) | 2.4 |  |
| PcTas3250-01 | Malignant | 1.06 | - | 1 (100%) | 1 | 3 |
|  | Benign | 1.08 | - | 1 (100%) | 1 |  |
| ^1^Transcribes (+) or does not transcribe (-) the variant G84E allele; ^2^Primer pair set used to assess CpG island methylation (as per Supplementary Figure 1); ^3^RNA extracted from a mixed cell population for assays. | | | | | | |

**Supplementary Table 3: Primers used for sequencing and RT-qPCR**

| **Method** | **Forward Primer (5’-3’)** | **Reverse Primer (5’-3’)** | **Amplicon Size (bp)** |
| --- | --- | --- | --- |
| *HOXB13* sanger sequencing (genomic DNA) | CACAACGGTCCCTCTTGTCT | GTTCAGCGGACGTAAGCG | 696 |
| *HOXB13* sanger sequencing (FFPE DNA) | CCGGATAGAAGGCAAACTCA | GCTGATGCCTGCTGTCAACT | 272 |
| *HOXB13 RT-qPCR* (Ma, 2004) | TTCATCCTGACAGTGGCAATAATC | CTAGATAGAAAATATGAGGCTAACGATCAT | 77 |
| *GAPDH RT-qPCR (Mori, 2008)* | CAACGGATTTGGTCGTATTGG | GCAACAATATCCACTTTACCAGAGTTAA | 72 |
| *β-Actin RT-qPCR (Mori, 2008)* | GAGCGCGGATACAGCTT | TCCTTAATGTCACGCACGATTT | 59 |
| *HOXB13* MiSeq sequencing (FFPE cDNA) | GGACACCGGCAGGAGTAGTA | CTGAGCCTGCTGTCAACT | 157 |
| Upstream CpG Island Methylation  (Product 1 in Supplementary Figure 1) | TTCTCCCAACTAAAACAAACTCTAT | GTAAAGGTTATAGGTTGTTTGTGGG | 254 |
| *HOXB13* Promoter/Exon 1 CpG Island Methylation (Liu, 2012)  (Product 2 in Supplementary Figure 1) | ACTTATTCTCTCTCTCTCTCT | CCTTAACTCCATCCAAAATAAC | 314 |
| *HOXB13* MiSeq Methylation  (Product 3 in Supplementary Figure 1) | TTAATTATGTTTTTTTGGATTTGTTAGGT | ACTACCTAAACACAAAATTTCAAC | 175 |
| *HOXB13* rs9900627 MiSeq sequencing | GGGAACCTACCAGCCTATGG | GTTCTGTTCTCCCTGGCAAC | 215 |
| Illumina adaptors used for MiSeq sequencing | TCGTCGGCAGCGTCAGATGTGTATAAGAGACAG | GTCTCGTGGGCTCGGAGATGTGTATAAGAGACAG | - |
